# Supplementary material for: Plasma-Functionalised Dressings for Enhanced Wound Healing
Source: Int J Mol Sci. 2023 Jan 2;24(1):797. doi: 10.3390/ijms24010797 (PMC9820862; doi:10.3390/ijms24010797)
Supplement: Supplementary file 1 [file ijms-24-00797-s001.zip › ijms-2048800-supplementary.pdf]

Supplementary Table S1.

Elements and Peak Fits	Binding Energy	Acrylic Acid	Allylamine	Melolin
O1s	532eV	29.4%	5.13%	21.17%
C1s	285eV	70.96%	78.54%	77.03%
N	399eV		15.22%	
F	688eV		1.11%	0.55%
Si	101eV			1.25%
C-C (Allylamine 1)	285eV	47.77%	69.6%	48.64%
C-OR + (/ C=N / C-N (Allylamine 2))	286.5eV	8.66%	23.93%	16.27%
C=OR	287.5eV	4.13%	4.38%	2.77%
C((=O)OR) + ((/RC(=O)NR (Allylamine 4)	288.9eV	19.73%	2.90%	12.23%
CC((=O)OR)	285.7eV	19.72%		12.25%
C-Si	284.1eV			7.84%
